# pTRA – A reporter system for monitoring the intracellular dynamics of gene expression

**DOI:** 10.1371/journal.pone.0197420

**Published:** 2018-05-17

**Authors:** Sabine G. Wagner, Martin Ziegler, Hannes Löwe, Andreas Kremling, Katharina Pflüger-Grau

**Affiliations:** Systems Biotechnology, Faculty of Mechanical Engineering, Technical University of Munich, Garching, Germany; University of Surrey, UNITED KINGDOM

## Abstract

The presence of standardised tools and methods to measure and represent accurately biological parts and functions is a prerequisite for successful metabolic engineering and crucial to understand and predict the behaviour of synthetic genetic circuits. Many synthetic gene networks are based on transcriptional circuits, thus information on transcriptional and translational activity is important for understanding and fine-tuning the synthetic function. To this end, we have developed a toolkit to analyse systematically the transcriptional and translational activity of a specific synthetic part *in vivo*. It is based on the plasmid pTRA and allows the assignment of specific transcriptional and translational outputs to the gene(s) of interest (GOI) and to compare different genetic setups. By this, the optimal combination of transcriptional strength and translational activity can be identified. The design is tested in a case study using the gene encoding the fluorescent mCherry protein as GOI. We show the intracellular dynamics of mRNA and protein formation and discuss the potential and shortcomings of the pTRA plasmid.

## Introduction

In the field of Synthetic Biology and bioengineering genetic parts with specific biological functions are manipulated or introduced into a host cellular system. For the successful implementation of such functions it is crucial to understand and predict the behaviour of synthetic genetic circuit itself and its impact on the host cell’s physiology [1,2]. Therefore, it is not only necessary to have good standards to measure and represent accurately the biological parts, but also to understand the interplay with the host’s cellular resources and functions. The conversion of a genetic sequence into a functional protein relies on the coordinated interplay of a multiplicity of biomolecules, which are provided by the host cell -at least until real orthogonallity is achieved [1]. Already good progress is made in the development of standards for genetic parts, as represented by the library of BioBricks and biological parts (http://partsregistry.org) with its extension by the iGEM students’ competition (http://www.igem.org), and the Standard European Vector Architecture (SEVA; http://seva.cnb.csic.es), a repository of formatted molecular tools [3,4]. At least equally important, is the description and parameterisation of biological functions [5]. As many synthetic gene networks involve transcriptional circuits, it is highly desirable to gain information on transcriptional and post-transcriptional events and their interconnection. As it is difficult to standardise these biological functions, it is important to characterise them with standardised methods. The most common methods for mRNA quantification require a complex protocol with the need of rapid quenching and are fastidious to run *in vivo*. Especially for pathway configurations involving autonomous components, it would be of advantage to have an easy-read-out assay with high-throughput capability to monitor the gene expression and to directly link this information to the amount of produced protein. The knowledge on the intracellular dynamics of transcription and translation of a specific genetic construct will contribute to the understanding of the interplay between the cellular resources provided by the host cell and the introduced gene. The understanding and predictability of the intracellular processes provide the basis for manipulating and fine-tuning the genetic circuit for optimal performance in the host cell’s environment. To this end, we have developed an *in vivo* and *on-line* measurable tool, which allows the systematic characterisation of transcriptional and translational output of specific constructs in engineered cells.

## Results and discussion

### Properties of the pTRA reporter plasmid

**Fig 1:**
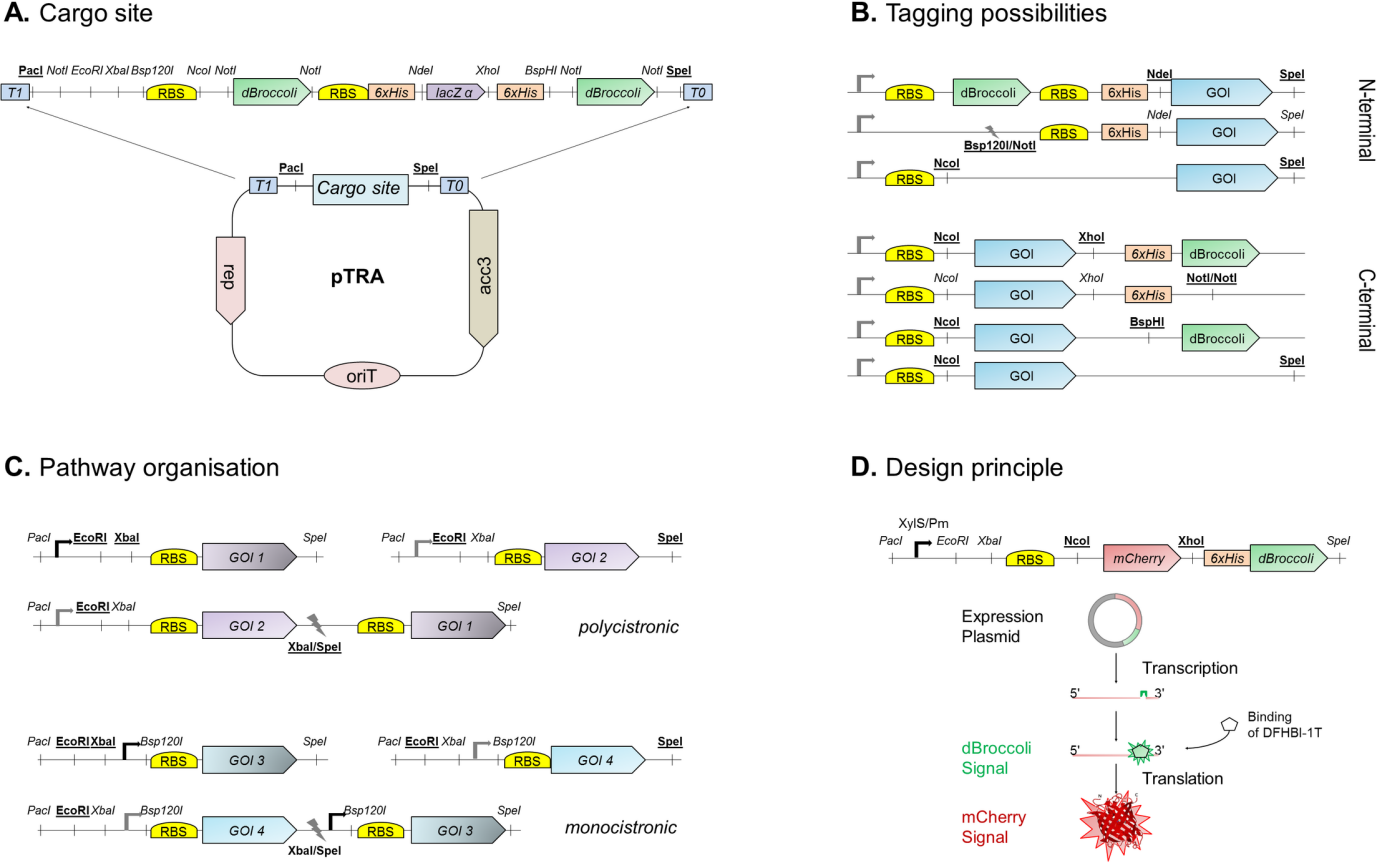
Structure and possibilities of pTRA for quantitative analysis of heterologous gene expression. (A) The multiple cloning site is designed to allow DNA assembly using the BioBrick method and compatible to the SEVA collection. Quantification of the transcriptional activity is enabled by the dBroccoli mRNA-tag, whereas the his-tag allows quantification of the translated protein. (B) The different possibilities for tagging the gene of interest (GOI) on either side are shown. (C) Depending on the restriction sites chosen, the gene clusters can be arranged in a mono- or polycistronic manner. (D) The plasmid pTRA-51hd used in this work. It carries the mCherry gene with the dBroccoli-tag fused to the 3’-end of the gene and the his-tag to the C-terminus of the protein. This plasmid was used for validation of the system, as it allows to follow mRNA synthesis by adding the dye DFHBI-1T to the cultures and protein formation by mCherry mediated fluorescence.

The centrepiece of the method is plasmid pTRA, which was designed for dynamic analysis of heterologous gene expression (Fig 1A). The transcriptional and translational activity can be followed by 5' or 3'-tagging of the mRNA with the F30-2xdBroccoli mRNA-tag [6] and N- or C-terminal his-tagging of the encoded protein [7]. The F30-2xdBroccoli mRNA-tag was chosen as it was proven that the F30-scaffold is highly effective for expressing aptamers in cells and prevents rapid degradation by endonucleases [8]. The organisation of the restriction sites of the multiple cloning site (MCS) of pTRA matches the SEVA standards and additionally allows DNA assembly using the BioBrick-method (Fig 1A and S1 Fig). This makes it a handy and versatile tool, which has the potential to be widely used to monitor the dynamics of transcription and translation of a given gene or pathway.

First, the promoter of choice has to be integrated into the plasmid. Depending on the user’s demands, genes can be organised polycistronically or a monocistronically following the BioBrick-concept for cloning (Fig 1C). This maintains the restriction sites *EcoRI*, *XbaI* and *SpeI* (which is compatible to *XbaI*) and allows shuffling of entire pathways. For a polycistronic configuration the promoter needs to be inserted with the enzymes *PacI* and *EcoRI*, whereas a monocistronic organisation of genes requires cloning via *XbaI* and *Bsp120I*.

Next, the position of the tags for the transcriptional and translational readout has to be defined. Cloning with *NdeI* and *SpeI* provides the dBroccoli-tag on the 5’-end of the mRNA transcript and the his-tag on the N-terminal part of the corresponding protein, whereas cloning with *NcoI* and *XhoI* places the tags on the opposite side (Fig 1B). We recommend tagging on the 3’-end of the mRNA to reduce the risk of negative influences on the transcription by the stable secondary structure of the F30-2xdBroccoli mRNA-tag. Such a double-label arrangement leads to his-tagging of the protein, which enables immuno-quantification and allows the fluorescent monitoring of the mRNA levels *in vivo*. At user's choice, it is also possible to select just one tag. An easy proof of successful cloning via blue-white-screening is provided since the *lacZα* gene is excised during the cloning process. We recommend using a *recA*^-^ strain for plasmid maintenance as the vector frame harbours a few repeated sequences. The broad variety of restriction sites within the multiple cloning site (MCS) widens the repertoire of pTRA by exchanging other parts of the system: the promoter, the RBS, or the nature of the mRNA-tag. This highly modular tagging-site combined with the pSEVA platform has the potential to provide individually tailored plasmids for nearly any expression analysis.

### Monitoring the dynamics of transcription and translation–a case study

**Fig 2:**
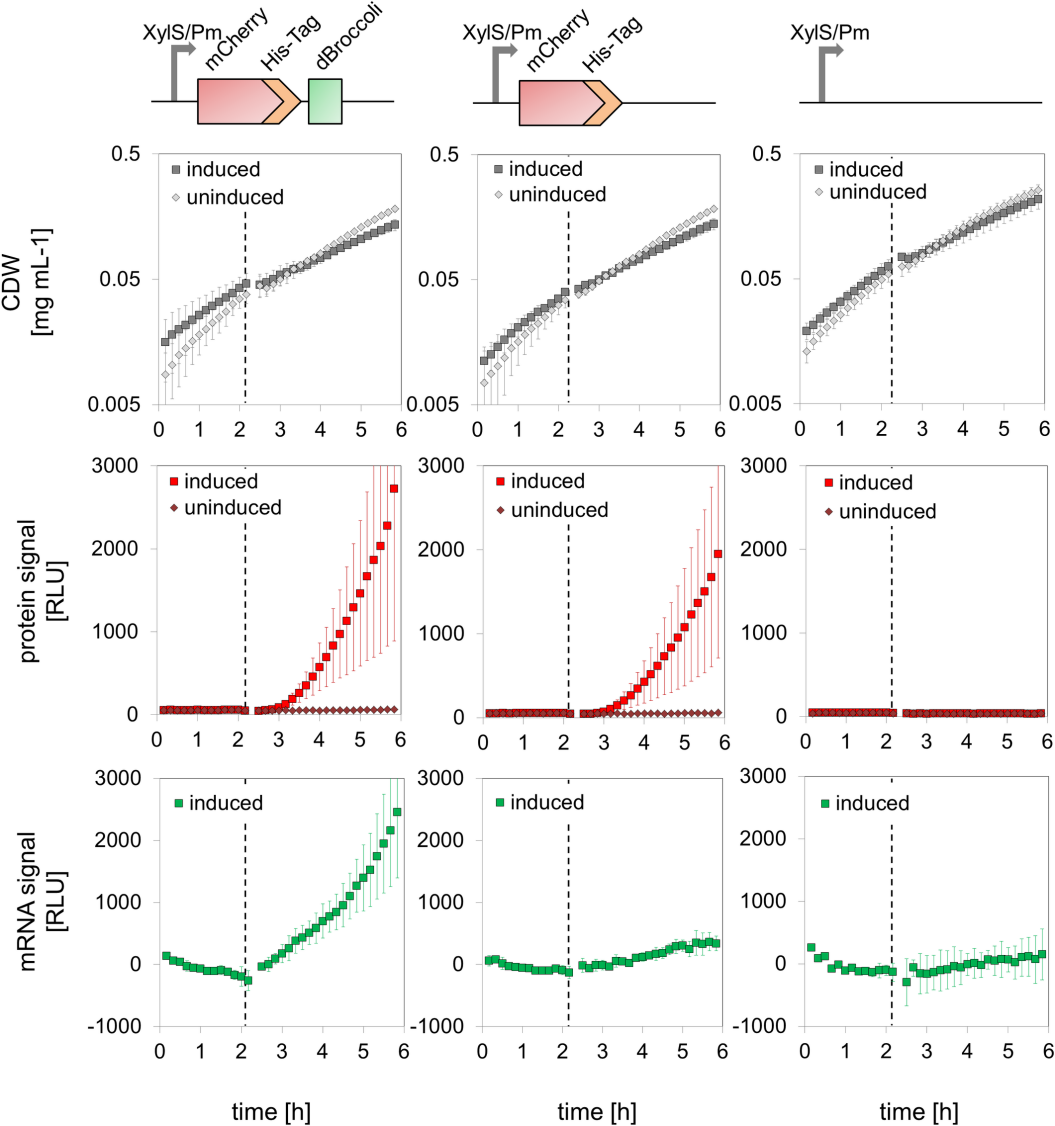
Transcriptional and translational output. Shown are the growth (upper row), protein signal (middle row) and mRNA signal (lower row) in cultures of *E*. *coli* HMS174/DE3 carrying pTRA-51hd (first column), pTRA-50 (second column, measured in duplicates), or pTRA-51h (last column). Cells were grown until the mid-logarithmic growth phase and induced (marked with dashed line) with m-Toluic acid (dark colour) or measured without induction (light colour). Shown are means and standard deviation from triplicates if not stated differently.

**Fig 3:**
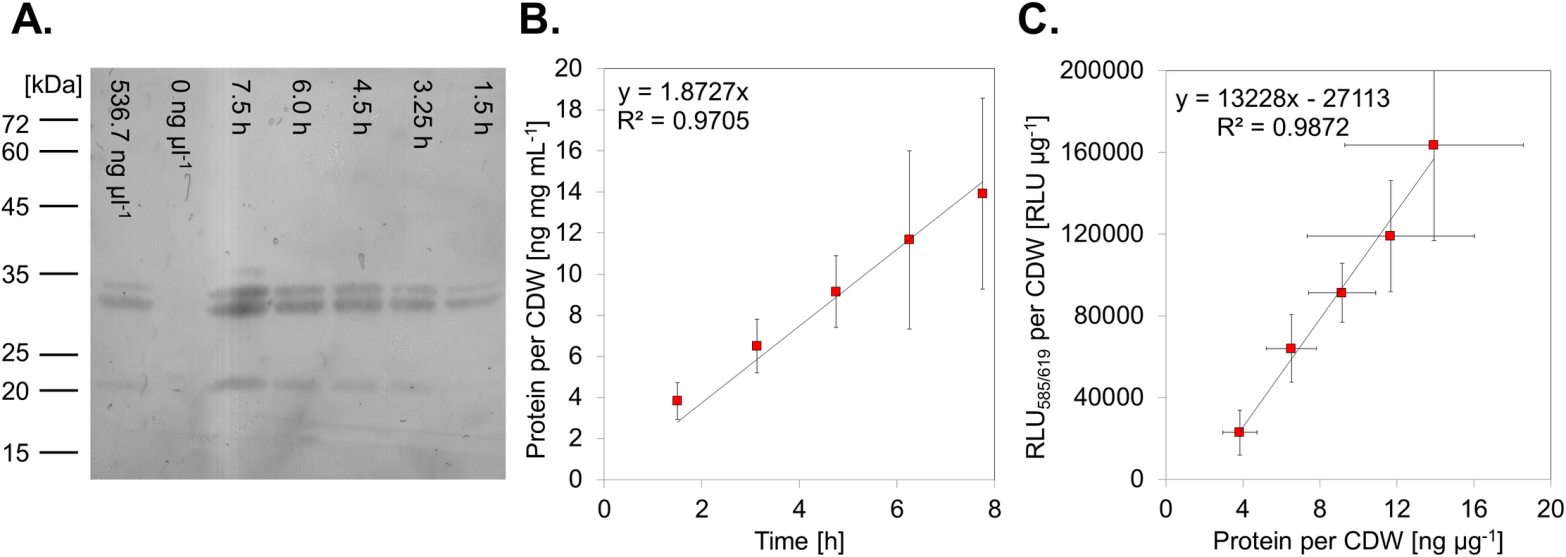
Immuno-quantification of mCherry. For quantification of the protein of interest (here mCherry) the his-tag was chosen. (A) Western blot with *α-*his antibody from samples taken at different time points after induction of gene expression (lanes 3–7). A two-point quantification (lane 1 and 2) was performed to match the intensities of the bands to a standard curve prepared with purified his-tagged mCherry (not shown). (B) Quantification of the intensities of the his-tag derived signal by determination of the peak area over time. (C) Correlation between the fluorescence derived signal and the immuno-quantified signal of the mCherry protein.

In order to show the potential of pTRA, we constructed pTRA-51hd (Fig 1D). As promoter the well-characterised XylS/Pm promoter was used [9,10]. As ribosome binding site (RBS) we chose the one that is used for the reporter genes of the SEVA database [4], and as the proxy of any gene of interest (GOI) the sequence encoding the fluorescent mCherry protein. This led to 3’-end tagging of the mRNA with the dBroccoli-tag [6] and C-terminal fusion of the his-tag to the mCherry protein. As schematically shown in Fig 1D, a GFP-like fluorescence signal reflecting the amount of mRNA is generated upon complex formation of the dye DFHBI-1T with the dBroccoli RNA-aptamer. We determined the time frame of dye uptake and signal formation and tested different dye concentrations for generating reliable measurements (S2 Fig). After 2 min a stable read out of the mRNA signal was achieved under all conditions tested. A dye concentration of 200 μM DFHBI-1T was sufficient to saturate the signal. In order to obtain data on the transcriptional and translational activity, we grew *E*. *coli* HMS174/DE3 (pTRA-51hd) in minimal medium and monitored the optical density (OD_600_), as well as green and red fluorescence (Fig 2). As controls we used the same *E*. *coli* strain, but with the corresponding empty plasmid pTRA-50 or a plasmid without the mBroccoli-tag (pTRA-51h) to correct for auto-fluorescence of the cells and check for effects of the RNA-tag on mRNA stability. Upon induction of transcription in all strains a slight reduction in the growth rate was observed, when compared to the uninduced control (Fig 2, upper row), even in the strain carrying the empty plasmid pTRA-50. We assigned this effect to the weak toxicity of the aromatic inducer. In the strains carrying a plasmid containing the mCherry gene (pTRA-51hd and pTRA-51h), red fluorescence was detectable 20 minutes after induction (Fig 2, middle row), reflecting the time necessary for transcription, translation, and maturation of the fluorescent protein [11]. Comparing the amount of protein produced between the different genetic setups, it can be observed that the cells carrying the dBroccoli-tagged version of the gene showed a slightly higher red fluorescent signal with a sharper increase than the ones without the RNA-tag. This suggests a stabilising effect of the dBroccoli-tag on the mRNA. This is in fact a welcome effect of our design, as this stabilisation should result in higher mRNA levels and might even allow studying otherwise rather unstable mRNAs. This potentially stabilising effect was observed in all experiments, even though the error bars resulting from calculating the mean of three different biological replicates are quite high. The high standard deviation is rather a result of biological variability than of plasmid instability, as it is only observed in cultures originating from different pre-cultures. If the replicates are derived from the same pre-culture, the absolute values are quite reproducible (see S3A Fig). The analysis of a sample of cultures in the late exponential growth phase by flow cytometry shows a uniform distribution of the fluorescent signals ruling out that a the variability in the fluorescent signal has its origin in the emergence of a sub-culture that has lost the plasmid (see Fig 3B).

To analyse the mRNA signal derived by binding of the dye DFHBI-1T to the dBroccoli-tagged mRNA (Fig 2, lower row), the raw fluorescent signal has to be processed to correct for autofluorescence of the cells and cell-density depended quenching of the dye DFHBI-1T. For a detailed description of the extraction of the dBroccoli signal see Material and Methods, S1 and S2 Files and S4 Fig.

Right after induction the mRNA signal clearly increases, showing that the amount of mRNA carrying the dBroccoli-tag increases. Again, the stabilising effect of the tag can be observed, as the signal steadily increases over time and does not reach a plateau. Cells carrying the empty plasmid as well as cells carrying the plasmid with the non-dBroccoli-tagged version do not show any obvious increase in the extracted dBroccoli signal along the growth. Thus, the dBroccoli tag of the pTRA plasmid seems to reflect the dynamics of mRNA transcript formation quite well. This information together with the information on the amount of produced protein now lays the basis for setting up and testing mathematical models describing the interplay of transcription and translation.

Since most GOIs will not have a fluorescent marker, we confirmed that quantification of the protein of interest is likewise possible by immuno-quantification (Fig 3) and correlates to the fluorescent output. We analysed samples taken at different time points after induction of transcription and determined the amount of his-tagged mCherry by hybridization with an anti-his antibody (Fig 3A). Quantification of the band intensities with a two-point calibration revealed a steady increase of the amount of mCherry protein produced (Fig 3B), which correlates well to the protein amount determined by fluorescent read-out (Fig 3C).

## Conclusion

**Fig 4:**
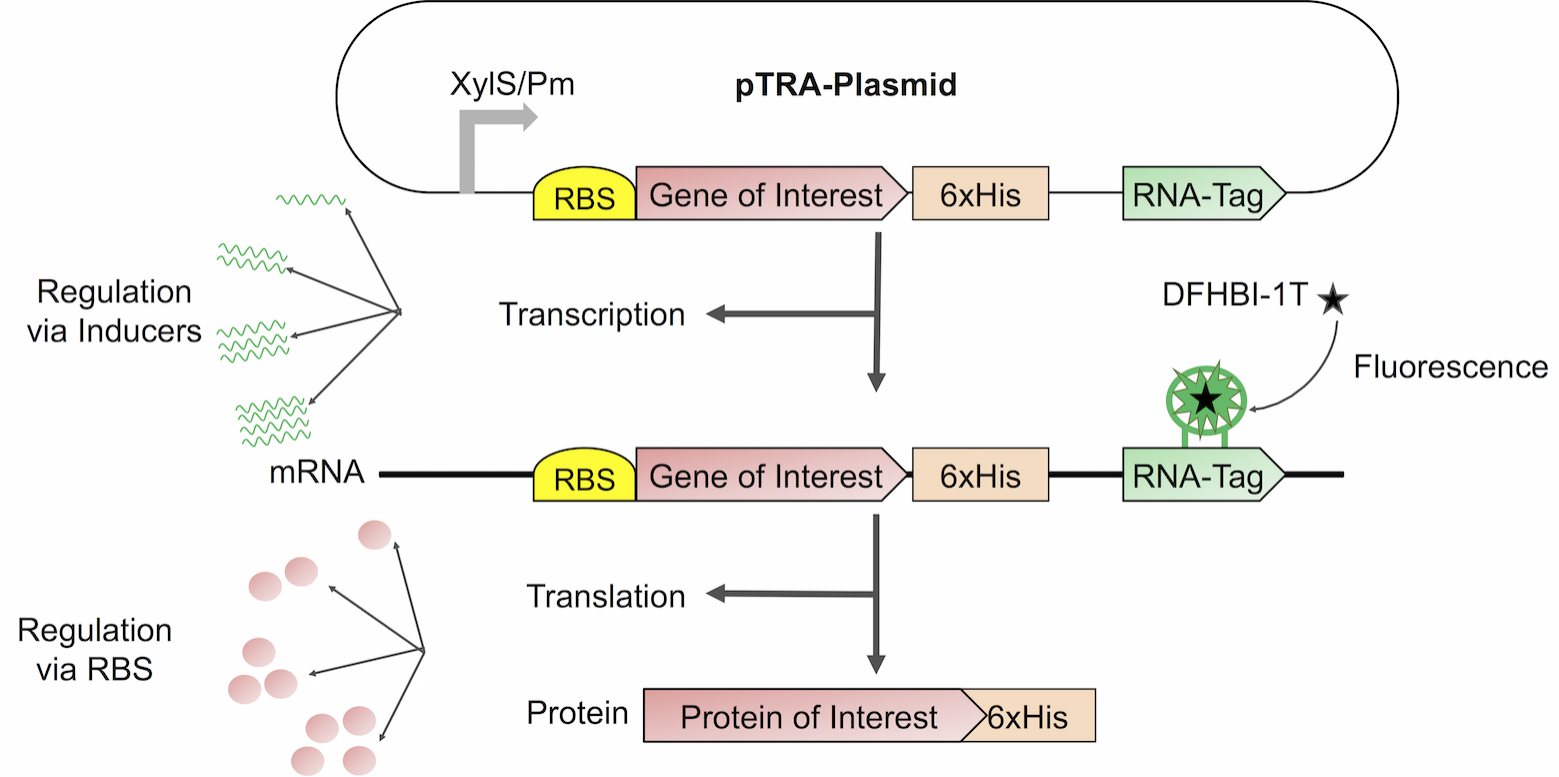
Possibilities of pTRA. By using different inducers and RBSs the amount of transcript and the translation rate can be determined. This would allow comparing the performance of different setups and fine-tuning of a synthetic pathway that is to be integrated into the cell.

Using various configurations of pTRA by combining different inducers to obtain altered transcription rates, and RBSs to vary the translation rate, allows for a thorough analysis of any heterologous gene or gene cluster in the respective system (Fig 4). It will be possible to assign specific activities to both, transcription and translation, and compare different genetic setups. By this, the optimal combination of transcriptional strength and translational activity can be identified. This information paves the way for tailored fine-tuning of any synthetic pathway that is to be integrated into the cell. In a future scenario, by the implementation of a mathematical model, the interplay of transcription and translation could be described in more detail and it might be possible to extract specific mRNA production rates. Thus, pTRA is a useful tool to determine the impact of transcription and translation on the overall protein outcome of a synthetic heterologous construct and will therefore contribute to assign and in the future to maybe even predict numbers for the specific activities.

## Materials and methods

### Cloning procedure

The plasmid pTRA was constructed by the insertion of a synthetic cargo sequence into pSEVA641 via the restriction sites *PacI* and *SpeI* (S1 Fig). The cargo sequence was ordered from BioCat GmbH (Heidelberg, Germany). To construct the plasmids pTRA-51hd, and pTRA-51h, first the XylS/Pm promoter had to be added. Therefore, pSEVA648 (carrying the XylS/Pm promoter) was digested with *EcoRI* and *SpeI* and the equally digested cargo site was inserted. It was necessary to introduce a silent mutation into the *NcoI* restriction site of the *xylS* sequence (C270A) via an overlap extension PCR performed with Primers SW16, SW28, SW29, and SW30 (Table 1), to enable *NcoI* based cloning of the gene of interest (resulting in plasmid: pTRA-50).

**Table 1:** Oligonucleotides used in this work.

Oligo-ID	Sequence (5’ - 3’)
SW16	gcctttcgttttatttgatgcct
SW22	gggggccatggaagtgagcaagggcgaggaggataa
SW23	gggggctcgagcttgtacagctcgtccatgcc
SW26	gtaatgcagaagaagactatgggctgggaggcct
SW27	aggcctcccagcccatagtcttcttctgcattac
SW28	aaaatagtgctcctggacatggccacgccacaggc
SW29	gcctgtggcgtggccatgtccaggagcactatttt
SW30	ttgggccctctagaggatcccc
SW68	tgatgactagttcagtggtggtggtggtggt

In order to clone the GOI, here mCherry, via *NcoI* and *XhoI*, it was first necessary to get rid of the intrinsic *NcoI* restriction site of the mCherry gene as well as to insert two additional bases to the 5’-end to avoid a frame shift. In brief, the mCherry sequence was amplified from pSEVA237R via an overlap extension PCR with the primers SW22, SW23, SW26, and SW27 (Table 1), *NcoI* and *XhoI* digested and ligated into the equally digested pTRA-50. This resulted in pTRA-51hd. As a control the plasmid pTRA-51h without the dBroccoli sequence was generated by generating a his-tagged mCherry gene with the oligonucleotides SW22 and SW68 (Table 1), that was inserted into pTRA-50 via *NcoI* and *SpeI*. In all cases, the plasmids were transformed into chemically competent *E*. *coli* HMS174/DE3 cells. Since the mRNA-Tag contains highly repetitive sequences *recA*^*-*^ strains are recommended for the storage of the plasmid. The sequence of pTRA has been deposited in the GeneBank under the accession number MG210576.

### Strains and cultivation

#### General growth conditions

All pre-cultures of *E*. *coli* cells were cultivated in Luria-Bertani (LB) medium [12]. The minimal medium was composed of a salt solution, amended with trace elements, and 20 mM of glucose as C- source. The pH was set to 7.4 (25°C) by the addition of 2.4 g L^-1^ NaOH to the salt solution [(NH_4_)_2_HPO_4_ 4 g L^-1^, KH_2_PO_4_ 13.3 g L^-1^] [13]. If necessary, the pH was additionally titrated with NaOH. Trace elements without C-source were prepared as described in Hiller *et al*. [14] with the difference that higher concentrations of CaCl_2_ (0.168 mM) and MgSO_4_ (2 mM) were used.

#### Microtiterplate experiments

Independent of the measurement taken, in all cases a general procedure was followed to prepare the cultures: First, cells of a single colony of *E*. *coli* HMS174/DE3 carrying pTRA-51hd, pTRA-51h, or pTRA-50, respectively, were inoculated in 5 mL LB-medium [12] and incubated at 37°C for maximally 8 hours. Of these exponentially growing cells, 50 μL were transferred to 5 mL minimal-medium. These intermediate cultures were incubated for 24 hours, until the stationary phase was reached. An aliquot was used to inoculate 5 mL minimal-medium to an OD_600_ of 0.05 (pre-culture). The next morning, these cultures were diluted with fresh minimal-medium to an OD_600_ of 0.02. Of this cell suspension 100 μL were transferred to a 96-well-plate and to each well another 100 μL of minimal-medium amended with either 2 μL of a 40 mM DFHBI-1T (Abcam, UK) in DMSO solution, or with an equal amount of solvent (2 μL DMSO). The microtiter plate was placed in an automated plate-reader (Infinite 200, Tecan) and incubated at 37°C. The optical density (Measurement Wavelength: 600 nm, Measurement Bandwidth: 9 nm, Number of Reads: 25, Settle Time: 0 ms) and fluorescence (green: 485/520 nm, red: 585/619 nm, Excitation Bandwidth: 9 nm, Emission Bandwidth: 20 nm, Reading Mode: Top, Lag Time: 0 μs, Integration Time: 20 μs, Number of Reads: 25, Settle Time: 0 ms, Gain Value: 100) were measured every 10 minutes. After each measurement the plate was shaken for 240 sec (Shaking Duration: 10 sec, Mode: Orbital, Amplitude: 3 mm, Frequency: 44.3 rpm). When the mid-exponential growth phase was reached (after 130 min of incubation), transcription was induced with 0.2 μL of m-Toluic acid (1.5 M, solved in DMSO). To the uninduced cells (inoculated from the same pre-culture) an equal amount of solvent was added (0.2 μL DMSO). Growth and fluorescence was followed another 3.5 h.

#### Samples for immuno-quantification

To obtain samples for immuno-quantification, pre-cultures were exactly prepared as described above. However, the next day they were used to inoculate 50 mL minimal medium to an OD_600_ of 0.05. Cultivation took place in unbaffled 250-mL-shaking-flasks at 250 rpm, and 37°C. In periodic intervals throughout the growth phase 2 mL samples were taken for Western blotting and immuno-quantification.

### Data processing

From the microtiterplate experiments the growth, the protein signal, and the mRNA signal were determined. If not stated differently, all experiments were performed in biological triplicates, i.e cultures derived from three different colonies. To assess growth, the optical density measured over time was background corrected with the corresponding value of the minimal medium without cells (supplemented with the same amount of additives). The optical density was converted to cellular dry weight applying the following correlation: CDW [mg mL^-1^] = 0.897*OD_600_. The growth rate is represented by the slope of the exponential fit and mCherry protein production was evaluated by following the red fluorescence (RLU585/619) over time.

The green fluorescence measured (RLU485/520) represents the sum of the autofluorescence of the cells and the actual dBroccoli signal. To extract the mRNA-signal from raw data (RLU485/520), two cultures inoculated with the same pre-culture were grown, one was induced and the other amended with an equal amount of inducer-solvent. To estimate the autofluorescence of the cells and to extract the mRNA signal a matlab script was designed (S3 File). For detailed information on how to prepare any data set and how to use the script please see S1–S3 Files and S4 Fig. In brief, the autofluorescence of the cells, which was determined from the non-induced culture and based on this data predicted for the induced culture, was subtracted from the green fluorescence of the induced culture, yielding the extracted dBroccoli signal over time.

### Western blotting and immuno-quantification

During cultivation 2 mL samples were taken in periodic intervals. Cells were pelleted at 16,800 x g for 10 min and diluted in 5x Laemmli buffer [15] to an OD_600_ of 5. After denaturation for 15 min at 95°C, 10 μL of the samples were loaded on a 12.5% SDS-PAGE. Separation took place at 300 V and 40 mA per gel for 45 min. Afterwards, the proteins were plotted to a nitrocellulose membrane (Whatman) for 20 min at 15 V with a semidry plotting chamber (Trans-Blot® SD Semi-Dry Transfer Cell, Bio-Rad Laboratories GmbH). After the blotting, membrane was blocked for 1 h at room temperature with 15 mL TBS-milk buffer (5% [w/v] milk powder, Tris 20 mM, NaCl 150 mM; pH 7.5). The membrane was incubated over night with 15 μL of the primary antibody (mouse anti-his antibody, Cat. No. 2-1590-001, IBA Göttingen) at 4°C. The blot was washed three times with 15 mL TBS-milk buffer, then 2.5 μL of the secondary antibody (Anti-mouse IgG, HRP-linked Antibody #7076, CST) were added. For signal detection the Opti-4CN detection kit (goat anti mouse, Biorad) was employed following the instructions of the provider. The plot was scanned with HP Scanjet 5470C (Hewlett-Packard Company) and the signal intensity was quantified from the scan with ImageJ. Shown are the results of three independent experiments.

### Nomenclature of pTRA plasmids

We recommend a systematic nomenclature, which contains all genetic components that are added to the pTRA cargo site. To shorten the plasmid names, we chose to number all used promoters, genes, and regulatory sites. Our nomenclature is based the style pTRA_Promoter_*d_h*_GOI_*h_d*, while *d* stands for dBroccoli-tag and *h* for his-tag. Unused features can be entitled as ‘0’. For example, the plasmid pTRA-51hd used in this work, is composed of the promoter ‘5’ (XylS/Pm), the gene of interest ‘1’ (mCherry), and the 3’-end tagging of the mRNA ‘d’ (dBroccoli-tag) as well as a C-terminal fusion ‘h’ (his-tag).

## Supporting information

S1 File Detailed description of data processing. (PDF)

S2 File Matlab script for extraction of the mRNA signal. (PDF)

S3 File Matlab file used in this work. (M)

S1 Fig Cargo site of pTRA. The pTRA cargo site is flanked by the termination sites T1 and T0 as recommended by the SEVA guidelines. The insertion of the promoter of choice should be performed using the restriction sites *PacI* and *Bsp120I*. The possibility to shuffle pathways following the BioBrick concept is provided by the restriction sites *EcoRI*, *XbaI*, and *SpeI*. Depending on the cloning strategy the mRNA tag F30-2xdBroccoli (green) and/or his-tag (orange) can be attached at the N- or C-terminus. A set of restriction sites enables the selective exchange of tags. Upon insertion of the gene of interest l*acZα* (purple) is removed, which enables blue- white screening. The ribosomal binding sites (yellow) are provided in the plasmid. The underlined sequences represent the restriction sites.
(PDF)

S2 Fig Parameter of mRNA online measurements with the dBroccoli-tag. (A) Time frame of signal generation. The duration of signal formation and uptake of DFHBI-1T was determined in a fully induced culture with 200 μM of the fluorophore DFHBI-1T. Note that after two minutes a stable read in mRNA signal out was generated. (B) Determination of the optimal dye concentration. The concentration of the fluorophore DFHBI-1T was tested in the range of 0 to 200 μM dye. Transcription of the pTRA-51hd cargo site was induced with three different inducers (m-Tuloic acid, p-Tuloic acid, and 3-Chlorobenzoic acid). Note that in either case the signal was saturated with 200 μM dye. (C) Time frame of transcription initiation. Transcription of mCherry with the dBroccoli- tag was induced with 1.5 mM m-Tuloic acid in a DFHBI-1T containing culture (200 μM) and the green fluorescence was determined every 30 sec in the first 10 min and with 2 min intervals until minute 32. Afterwards fluorescence was measured in intervals of 5 min.
(PDF)

S3 Fig Variability of the mRNA and protein signals. (A) The extracted mRNA as well as the mCherry signals are highly reproducible, if the same experiment is performed with several different cultures (here seven) inoculated from the same pre-culture (left column). In contrast, if the signals from three independent cultures inoculated from three different pre-cultures are analysed, a high standard deviation is observed (right column), pointing towards a biologically determined variability. (B) Fluorescence distribution of the wild type and a pTRA_51hd carrying *E*. *coli* strain as determined by flow cytometry in the late exponential growth phase. Note, that the fluorescence distribution is uniform and no sub-population is detectable, as it would be expected if the plasmid was unstable.
(PDF)

S4 Fig Workflow of mRNA signal extraction. (A) Data organisation, (B) data integration into the Matlab scipt, (D) prediction of the autofluorescence of the test strain based on the control strain, and (D) mRNA signal extraction. See text for a detailed description of data processing and the Matlab script.
(PDF)
