# Supplementary material for: pTRA – A reporter system for monitoring the intracellular dynamics of gene expression
Source: PLoS One. 2018 May 17;13(5):e0197420. doi: 10.1371/journal.pone.0197420 (PMC5957375; doi:10.1371/journal.pone.0197420)
Supplement: S1 File — (PDF) [file pone.0197420.s001.pdf]

S1 File. Detailed description of data processing

To assess growth, the optical density measured over time was background corrected with the corresponding value of the minimal medium without cells (supplemented with the same amount of additives). The growth rate is represented by the slope of the exponential fit and mCherry protein production was evaluated by following the red fluorescence (RLU585/619) over time.

The green fluorescence measured (RLU485/520) represents the sum of the autofluorescence of the cells and the actual dBroccoli signal. To extract the mRNA-signal from raw data (RLU485/520), two cultures inoculated with the same preculture were grown, one was induced and the other amended with an equal amount of inducer-solvent. To estimate the autofluorescence of the cells and to extract the mRNA signal a Matlab script was designed. To use the script for a certain set of data, some parameters have to be adjusted as described in the following. For an overview of the procedure see Fig S4.

1. The data set is organised as depicted in Fig. S4A.
2. In the Matlab script the ‘xxx’ is replaced with these data-matrixes. The blanked optical density is inserted in OD=[xxx] and the green fluorescence values (RLU485/520) are copied to RLU485_520=[xxx]. The corresponding time points have to be introduced in vector form (see Fig. S4B, Time=[xxx]).
3. As constraints, the first and the last time point of interest, as well the induction time point and the number of replicates are be filled in.
   - First of all, the number of replicates (here 3) are added to the Matlab script:
     ```
     number_of_replicates=[xxx]; %replicates
     ```
   - The time points of interest are defined:
     ```
     start_point= xxx;%first time point of interest [h]
     induction_time_point=xxx;%first time point after induction [h]
     end_point=xxx;%last time point of interest [h]
     ```
   - Optionally: A OD to CDW correlation can be inserted in the following layout: `CDW [mg mL-1]=a*OD+b.` Otherwise state CDW=OD
   - The culture of interest is chosen (e.g. culture 1 out of 3 replicates). Note: each culture has to be analysed separately.
     ```
     clone_number=xxx;%choose replicate that should be analysed
     ```
4. The script is run. As the autofluorescence can be influenced by the addition of solvents or other chemical additives, the data is split up into two phases: A pre- and a post-inductional section. To evaluate the evolution of autofluorescence over time, the RLU485/520 signal of the control is analysed against the CDW. The both phases are fitted separately by a two term exponential model (f(x) describes the pre-induction period, g(x) is the fit-function for post-induction data) (Fig. S4C). This type of stretched exponential function was also applied by Lee et al. [1] to describe signals that depend on intracellular components like tryptophan. Two term exponential functions are able to describe signals consisting of two components. In this case here, one part of the signal is caused by the cellular autofluorescence and the other factor is the quenching of the dye fluorescence by the cell density.

5. Based on the functions for the fit, f(x) and g(x), which are determined by using the non-induced control, the autofluorescence of the test-strain for each CDW is predicted. The predicted autofluorescence is subtracted from the measured green fluorescence (Fig. S4D), yielding the extracted mRNA signal. This is found in the workspace as vector *'calculated_Broccoli'*.

Reference

1. Lee KC, Siegel J, Webb SE, Lévêque-Fort S, Cole MJ, Jones R, et al. Application of the stretched exponential function to fluorescence lifetime imaging. Biophysical Journal. 2001;81: 1265–1274. doi:10.1016/S0006-3495(01)75784-0
