# Supplementary material for: pTRA – A reporter system for monitoring the intracellular dynamics of gene expression
Source: PLoS One. 2018 May 17;13(5):e0197420. doi: 10.1371/journal.pone.0197420 (PMC5957375; doi:10.1371/journal.pone.0197420)
Supplement: S2 File — (PDF) [file pone.0197420.s002.pdf]

S2 File. Matlab script for extraction of the mRNA signal

```
clear all; close all;

%order of data (dye,inducer):
number_of_replicates=xxx;%replicates
position_of_control=1;%control=(+,-)
position_of_test_strain=2;%test strain=(+,+)

start_point=xxx;%first time point of interest [h]
induction_time_point=xxx;%first time point after induction [h]
end_point=xxx;%last time point of interest [h]
clone_number=xxx;%choose replicate that should be analysed

%add data
%avoid matrix dimension mismatch and replace ‘overflow’ with NaN
Time=[xxx];
OD=[xxx];
RLU485_520=[xxx];

%identification of relevant position in data matrix
i=number_of_replicates-clone_number;
%control w/o dye w/o induction
control=[position_of_control*number_of_replicates-i];
%data with dye with induction
dataset=[position_of_test_strain*number_of_replicates-i];

x=find(Time>=start_point,1);
y=find(Time>=induction_time_point,1);
z=find(Time>=end_point,1);

%add your CDW-OD correlation CDW [mg mL-1]=a*OD+b
%to use OD values write CDW=OD
CDW=OD

%%%GREEN FLUORESCENCE CAUSED BY AUTOFLUORESCENCE AND QUENCHING%%%
calculated_Broccoli= RLU485_520(dataset,x:z)

%uninduced
    %f(x) = a*exp(b*x) + c*exp(d*x)
    f = fit(CDW(control,x:y-1)', RLU485_520(control,x:y-1)', 'exp2');
    [fitresult, gof]=fit(CDW(control,x:y-1)', RLU485_520(control,x:y-1)',f
);
%Fit model to data
    %Parameters of fit
    f1=f.a;
    f2=f.b;
    f3=f.c;
    f4=f.d;
calculated_Autofluorescence=f1*exp(f2*CDW(dataset,x:y-
1))+f3*exp(f4*CDW(dataset,x:y-1));
calculated_Broccoli(1:(y-x))=(calculated_Broccoli(1:(y-x))-
calculated_Autofluorescence);

%induced
    %f(x) = a*exp(b*x) + c*exp(d*x)
    g = fit(CDW(control,y:z)', RLU485_520(control,y:z)', 'exp2');
    [fitresult, gof] = fit( CDW(control,y:z)', RLU485_520(control,y:z)',f);
    % Fit model to data
    %Parameters of fit
```

```
    g1=g.a;
    g2=g.b;
    g3=g.c;
    g4=g.d;
calculated_Autofluorescence=
g1*exp(g2*CDW(dataset,y:z))+g3*exp(g4*CDW(dataset,y:z));
calculated_Broccoli(y-x+1:z-x+1)=(calculated_Broccoli(y-x+1:z-x+1)-
calculated_Autofluorescence)

%calculated Broccoli signal over time
figure
plot(Time(x:z), calculated_Broccoli);
xlabel('Time, [h]');
ylabel('calculated Broccoli, [a.u.]');
%export_fig 'path\name.filetype' -nocrop -transparent

%fit of autofluorescence
figure
plot(f, CDW(control,x:y), RLU485_520(control,x:y));
xlabel('CDW, [mg mL-1]');
ylabel('RLU485/520, [a.u.]');
hold
plot(g, CDW(control,y:z), RLU485_520(control,y:z));
xlabel('CDW, [mg mL-1]');
ylabel('RLU485/520, [a.u.]');
%export_fig 'path\name.filetype' -nocrop -transparent

%solution can be found in workspace as 'calculated_Broccoli'
%Figures can be exported via the plug-in export_fig
```
