# Supplementary material for: pTRA – A reporter system for monitoring the intracellular dynamics of gene expression
Source: PLoS One. 2018 May 17;13(5):e0197420. doi: 10.1371/journal.pone.0197420 (PMC5957375; doi:10.1371/journal.pone.0197420)
Supplement: S1 Fig — The pTRA cargo site is flanked by the termination sites T1 and T0 as recommended by the SEVA guidelines. The insertion of the promoter of choice should be performed using the restriction sites PacI and Bsp120I. The possibility to shuffle pathways following the BioBrick concept is provided by the restriction sites EcoRI, XbaI, and SpeI. Depending on the cloning strategy the mRNA tag F30-2xdBroccoli (green) and/or his-tag (orange) can be attached at the N- or C-terminus. A set of restriction sites enables the selective exchange of tags. Upon insertion of the gene of interest lacZα (purple) is removed, which enables blue- white screening. The ribosomal binding sites (yellow) are provided in the plasmid. The underlined sequences represent the restriction sites. (PDF) [file pone.0197420.s004.pdf]

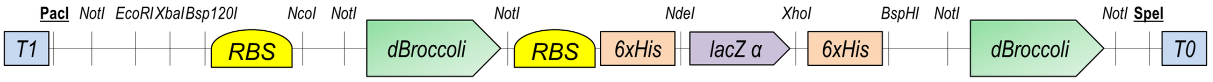

PacI NotI EcoRI XbaI Bsp120I NcoI NotI
TTAATTAAAGCGGATAACAATTTCACACAGGAGGCCGCCTAGGCCGCGGCCGCGCGAATTCGAGCTCGGTACCCGGGGATCCTCTAGAGGGCCCAAGAAGGAGATATACCATGGAAAGCGGCCGC

TTGCCATGTGTATGTGGGAGACGGTCGGGTCCATCTGAGACGGTCGGGTCCAGATATTCGTATCTGTCGAGTAGAGTGTGGGCTCAGATGTCGAGTAGAGTGTGGGCTCCCACATACTCTGAT

NotI
GATCCAGACGGTCGGGTCCATCTGAGACGGTCGGGTCCAGATATTCGTATCTGTCGAGTAGAGTGTGGGCTCAGATGTCGAGTAGAGTGTGGGCTGGATCATTCATGGCAATGAGCGGCCGC

NdeI
AAGAAGGAGATATACCATGAGCAGCAGCCATCATCATCATCATCACCATATGGGCGATCCGAAACTGCTGCTGGAAGTGCCGCATGCGATTAGCAGCAGCCCGGGCAACAGCCTGGCGGTGGT

GCTACAGCGCCGCGATTGGGAAAACCCGGGCGTGACCCAGCTGAACCGCCTGGCGGCGCATCCGCCGTTTGCGAGCTGGCGCAACAGCGAAGAAGCGCGCACCGATCGCCCGAGCCAGC

XhoI BspHI NotI
AGCTGCGCAGCCTGATTCGCCTGCTGACCAAACCGGAACGCAAACTGAGCTGGCTGCTGCCGCCGCTGAGCAACAACTGACTCGAGCACCACCACCACCACCACTGATCATGAGGCGGCCGC

TTGCCATGTGTATGTGGGAGACGGTCGGGTCCATCTGAGACGGTCGGGTCCAGATATTCGTATCTGTCGAGTAGAGTGTGGGCTCAGATGTCGAGTAGAGTGTGGGCTCCCACATACTCTGAT

NotI
GATCCAGACGGTCGGGTCCATCTGAGACGGTCGGGTCCAGATATTCGTATCTGTCGAGTAGAGTGTGGGCTCAGATGTCGAGTAGAGTGTGGGCTGGATCATTCATGGCAATGAGCGGCCGC

SpeI
TTGACTAGT

S1 Fig: Cargo site of pTRA. The pTRA cargo site is flanked by the termination sites T1 and T0 as recommended by the SEVA guidelines. The insertion of the promoter of choice should be performed using the restriction sites *PacI* and *Bsp120I.* The possibility to shuffle pathways following the BioBrick concept is provided by the restriction sites *EcoRI*, *XbaI*, and *SpeI.* Depending on the cloning strategy the mRNA tag F30-2xdBroccoli (green) and/or his-tag (orange) can be attached at the N- or C-terminus. A set of restriction sites enables the selective exchange of tags. Upon insertion of the gene of interest *lacZα* (purple) is removed, which enables blue-white screening. The ribosomal binding sites (yellow) are provided in the plasmid. The underlined sequences represent the restriction sites.
