# Supplementary material for: pTRA – A reporter system for monitoring the intracellular dynamics of gene expression
Source: PLoS One. 2018 May 17;13(5):e0197420. doi: 10.1371/journal.pone.0197420 (PMC5957375; doi:10.1371/journal.pone.0197420)
Supplement: S2 Fig — (A) Time frame of signal generation. The duration of signal formation and uptake of DFHBI-1T was determined in a fully induced culture with 200 μM of the fluorophore DFHBI-1T. Note that after two minutes a stable read in mRNA signal out was generated. (B) Determination of the optimal dye concentration. The concentration of the fluorophore DFHBI-1T was tested in the range of 0 to 200 μM dye. Transcription of the pTRA-51hd cargo site was induced with three different inducers (m-Tuloic acid, p-Tuloic acid, and 3-Chlorobenzoic acid). Note that in either case the signal was saturated with 200 μM dye. (C) Time frame of transcription initiation. Transcription of mCherry with the dBroccoli- tag was induced with 1.5 mM m-Tuloic acid in a DFHBI-1T containing culture (200 μM) and the green fluorescence was determined every 30 sec in the first 10 min and with 2 min intervals until minute 32. Afterwards fluorescence was measured in intervals of 5 min. (PDF) [file pone.0197420.s005.pdf]

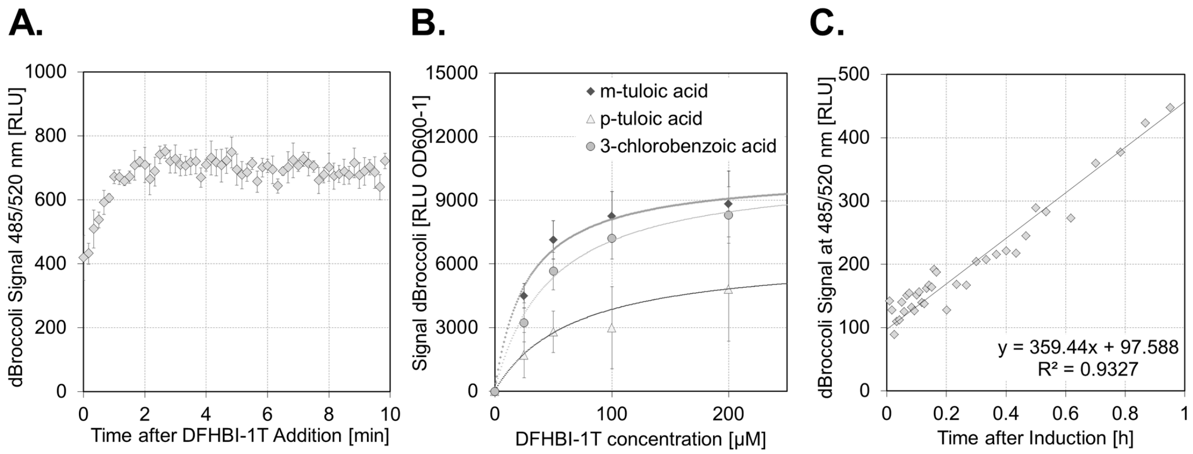

S2 Fig: Parameter of mRNA online measurements with the dBroccoli-tag. (A) Time frame of signal generation. The duration of signal formation and uptake of DFHBI-1T was determined in a fully induced culture with 200 µM of the fluorophore DFHBI-1T. Note that after two minutes a stable read in mRNA signal out was generated. (B) Determination of the optimal dye concentration. The concentration of the fluorophore DFHBI-1T was tested in the range of 0 to 200 µM dye. Transcription of the pTRA-51hd cargo site was induced with three different inducers (m-Tuloic acid, p-Tuloic acid, and 3-Chlorobenzoic acid). Note that in either case the signal was saturated with 200 µM dye. (C) Time frame of transcription initiation. Transcription of mCherry with the dBroccoli-tag was induced with 1.5 mM m-Tuloic acid in a DFHBI-1T containing culture (200 µM) and the green fluorescence was determined every 30 sec in the first 10 min and with 2 min intervals until minute 32. Afterwards fluorescence was measured in intervals of 5 min.
