# Supplementary figures and images for: pTRA – A reporter system for monitoring the intracellular dynamics of gene expression
Source: PLoS One. 2018 May 17;13(5):e0197420. doi: 10.1371/journal.pone.0197420 (PMC5957375; doi:10.1371/journal.pone.0197420)

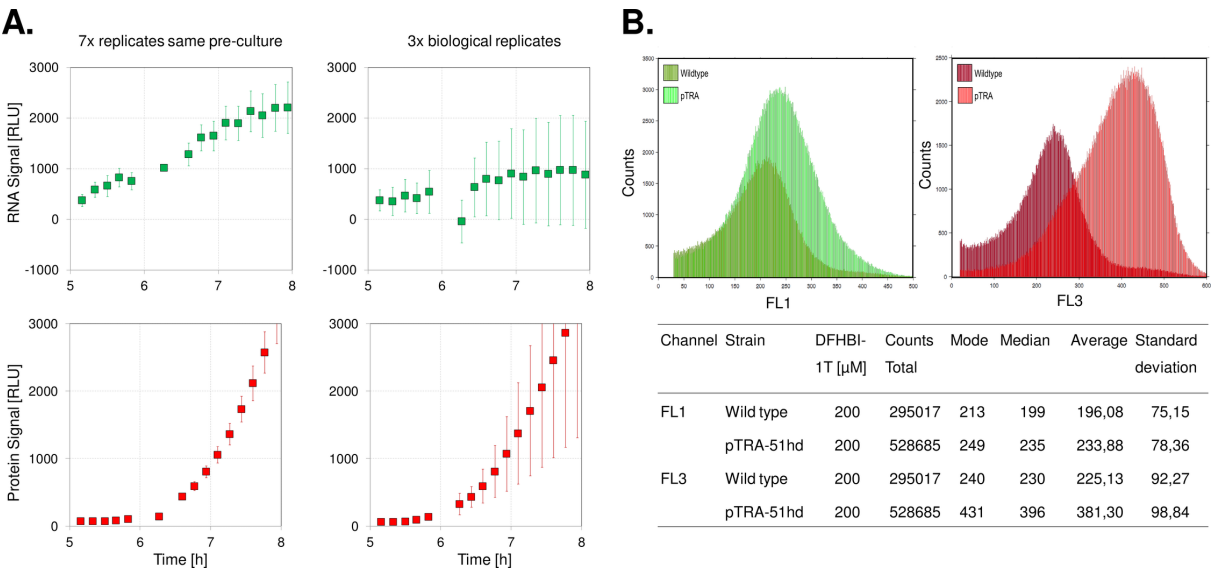

Supplement: S3 Fig — (A) The extracted mRNA as well as the mCherry signals are highly reproducible, if the same experiment is performed with several different cultures (here seven) inoculated from the same pre-culture (left column). In contrast, if the signals from three independent cultures inoculated from three different pre-cultures are analysed, a high standard deviation is observed (right column), pointing towards a biologically determined variability. (B) Fluorescence distribution of the wild type and a pTRA_51hd carrying E. coli strain as determined by flow cytometry in the late exponential growth phase. Note, that the fluorescence distribution is uniform and no sub-population is detectable, as it would be expected if the plasmid was unstable. (PDF) [file pone.0197420.s006.pdf]
