# Supplementary material for: pTRA – A reporter system for monitoring the intracellular dynamics of gene expression
Source: PLoS One. 2018 May 17;13(5):e0197420. doi: 10.1371/journal.pone.0197420 (PMC5957375; doi:10.1371/journal.pone.0197420)
Supplement: S4 Fig — (A) Data organisation, (B) data integration into the Matlab scipt, (D) prediction of the autofluorescence of the test strain based on the control strain, and (D) mRNA signal extraction. See text for a detailed description of data processing and the Matlab script. (PDF) [file pone.0197420.s007.pdf]

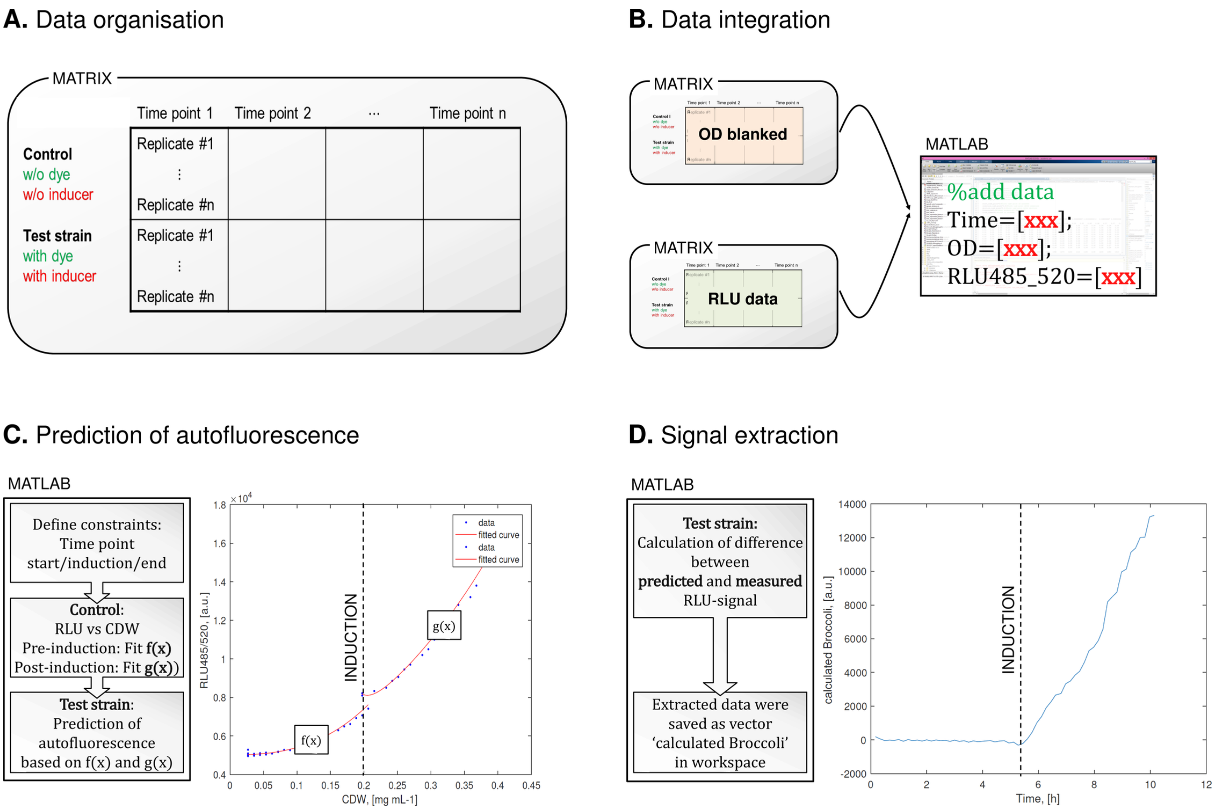

S4 Fig: Workflow of mRNA signal extraction. (A) Data organisation, (B) data integration into the Matlab scipt, (D) prediction of the autofluorescence of the test strain based on the control strain, and (D) mRNA signal extraction. See text for a detailed description of data processing and the Matlab script.
